# BioVDB: biological vector database for high-throughput gene expression meta-analysis

**DOI:** 10.3389/frai.2024.1366273

**Published:** 2024-03-08

**Authors:** Michał J. Winnicki, Chase A. Brown, Hunter L. Porter, Cory B. Giles, Jonathan D. Wren

**Affiliations:** ^1^Genes and Human Disease Research Program, Oklahoma Medical Research Foundation, Oklahoma City, OK, United States; ^2^Oklahoma Center for Neuroscience, University of Oklahoma Health Sciences Center, Oklahoma City, OK, United States; ^3^Department of Biochemistry and Molecular Biology, University of Oklahoma Health Sciences Center, Oklahoma City, OK, United States; ^4^Oklahoma Nathan Shock Center, Oklahoma City, OK, United States

**Keywords:** gene expression database, vector database, data mining, Gene Expression Omnibus, meta-analysis, Artificial Intelligence, Deep Learning

## Abstract

High-throughput sequencing has created an exponential increase in the amount of gene expression data, much of which is freely, publicly available in repositories such as NCBI's Gene Expression Omnibus (GEO). Querying this data for patterns such as similarity and distance, however, becomes increasingly challenging as the total amount of data increases. Furthermore, vectorization of the data is commonly required in Artificial Intelligence and Machine Learning (AI/ML) approaches. We present BioVDB, a vector database for storage and analysis of gene expression data, which enhances the potential for integrating biological studies with AI/ML tools. We used a previously developed approach called Automatic Label Extraction (ALE) to extract sample labels from metadata, including age, sex, and tissue/cell-line. BioVDB stores 438,562 samples from eight microarray GEO platforms. We show that it allows for efficient querying of data using similarity search, which can also be useful for identifying and inferring missing labels of samples, and for rapid similarity analysis.

## 1 Introduction

High-throughput sequencing data is being generated in large amounts, Reuter et al. (2015) creating an exponential increase in experimental gene expression data (Giles et al., 2017). Millions of freely accessible -omic samples are being deposited in public biological databases, and are an invaluable source of information for meta-analysis of gene expression networks, which can be used to predict gene function (Wren, 2009) or identify novel biomarkers of various disease phenotypes (Griffith et al., 2006). Simultaneously with the increase in high-throughput sequencing data, there has been an increase in the popularity of Machine Learning (ML) and Deep Learning (DL) models in biomedical sciences, which typically require massive data sets (Martorell-Marugán et al., 2019). Meta-analytical approaches can aid in the replication of results and provide increased statistical power and external validity. However, to effectively use experimental data and AI models, it is desirable to create a database in a standardized format amenable to such algorithms, which will provide easy querying and retrieval of the analyzed samples.

There are many repositories for transcriptional data, such as NCBI's Gene Expression Omnibus (GEO; Clough and Barrett, 2016), EBI ArrayExpress (Sarkans et al., 2020), and Sequence Read Archive (SRA; Katz et al., 2021). GEO alone, at the time of writing this manuscript, contains more than 6.6 million samples from over 25,000 technological platforms and over 200,000 experiments, and is rising exponentially (Supplementary Figure 1). It also archives gene expression experimental data derived from various types of assays (Supplementary Table 1). The data being available is only the first step in the process. Performing meta-analyzes also requires processing natural language into tabular annotations and standardization of independently-performed experiments, which is a challenge of its own (Hawkins et al., 2022). Further, the samples may be annotated well for a particular study (e.g., Alzheimer's status), but lacking other important annotations for meta-analyzes (e.g., Diabetes status in those same Alzheimer's samples). In addition to the sample-level difficulties mentioned above, there are also platform and data-type challenges to overcome. Multiple platforms contain information about gene expression, but these may be stored as raw or pre-transformed values in the matrices processed by each investigator. Microarrays can alleviate this problem, as we can re-derive the expression values from the raw data efficiently, but there is no obvious best solution for sequencing. The annotation of genes is also difficult when approaching cross-platform problems; array experiments contain probes that must be coerced into a common gene annotation space from a mixture of probes covering different gene segments. The bioinformatics community has responded to these needs in part (Hruz et al., 2008; Cheng et al., 2010; Lakiotaki et al., 2018; Franzén et al., 2019). However, none of these resources provide a vectorized data format that is AI-amenable, which would greatly facilitate the use of these databases for end-users.

A vector database (VDB) is a specialized type of database that is based on storing data in the form of a sequence of numbers—vectors. In this data storage format, each data point is encoded in a multidimensional vector space along with its metadata as an optional object associated with it. Several vector databases/vector search engines such as Pinecone (https://www.pinecone.io/), Weaviate (https://weaviate.io/), ChromaDB (https://www.trychroma.com/), Milvus (Wang et al., 2021), and Qdrant (https://qdrant.tech/) are already available. Even the once popular MongoDB recently released the Atlas Vector Search tool (https://www.mongodb.com/products/platform/atlas-vector-search), which allows semantic database searching. These recent examples suggest a new trend in the field. Importantly, VDBs significantly differ in schema compared to traditional databases. In the case of databases organized in a tabular format, searching is based on finding a given value in specific columns. In contrast, VDBs, after creating a query, search data points through similarity search, using distance metrics such as Euclidean distance, dot product, or cosine similarity (Taipalus, 2023). Moreover, such retrieved data is in a ready-to-use format for training Deep Learning models. In terms of AI applications, VDBs offer improved performance, reduced latency and function with billions of data points, allowing us to scale to most meta-analytical needs. With these features, such a data storage format has many advantages for use in microarray or sequencing experiments where experimental data often benefits from being represented as annotated vectors, allowing quick comparison across tissues, cell types, and disease states. Leveraging the unique features of VDBs to store inherently multidimensional biological data, and be compatible with state-of-the-art neural network architectures, stands to greatly benefit bioinformatics and biomedical sciences in general.

## 2 Methods

The graphical summary of our framework is shown in Graphical Abstract.

**Graphical Abstract:**
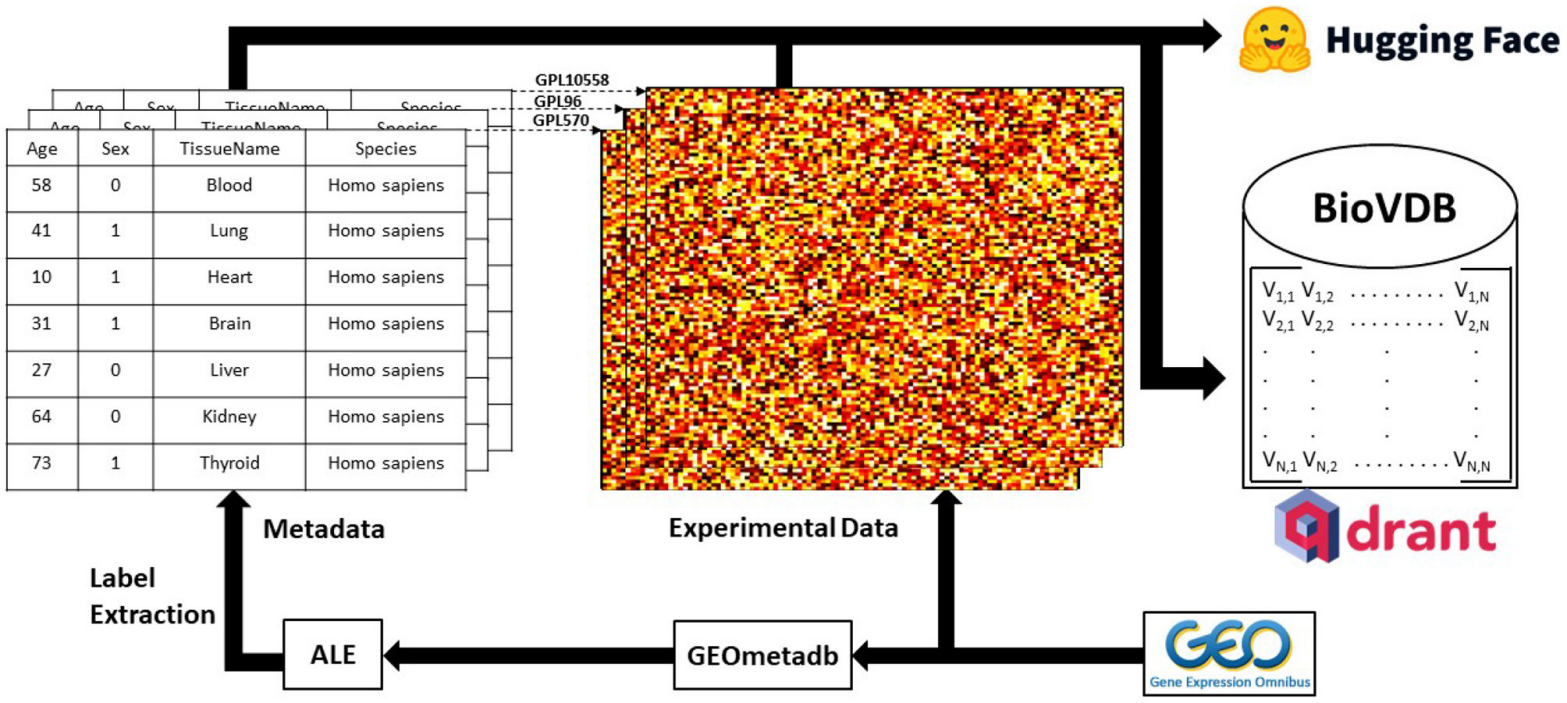
Graphical summary of the research framework. Experimental raw data was downloaded from GEO and metadata labels were extracted using ALE (Giles et al., 2017) on GEOmetadb R package (Zhu et al., 2008). Qdrant (https://qdrant.tech/) was used as a vector search engine for the database. Complete datasets are available at: https://huggingface.co/collections/mwinn99/biovdb-658daf0c3ceccd00f3ad63a9.

### 2.1 Gene expression data

Experimental gene expression data, for *Homo sapiens* and *Mus musculus* were downloaded from GEO. For *Homo sapiens* we currently include 335,962 samples from the GPL570, GPL96, GPL10558, GPL6947 platforms, and for *Mus musculus* we include 102,600 samples from the GPL1261, GPL6885, GPL6887, and GPL7202 platforms. Probes were collapsed to gene-level, based on average expression per gene, using official gene symbols associated with Entrez Gene ID. Experimental expression values are in raw format, in order to provide freedom in the context of choosing the method of data normalization for end users that best suits their needs.

### 2.2 Metadata

The metadata was obtained through the use of automated label extraction software (ALE; Giles et al., 2017), which relies on heuristic algorithms like string-matching and regular expressions approaches for labels extraction from GEO textual metadata. It contains information about the platform and experiment from which the sample came, as well as the age, sex, tissue, and organism of origin. All samples stored in the BioVDB are described in a uniform way. The “GSM” field contains the name of the particular GEO GSM sample (e.g., GSM1003121). In the same way, the “PlatformID” field describes the GEO GPL platform that was used in a given experiment (e.g., 10,558 stands for GPL10558), and the “ExperimentID” field contains the name of the GEO GSE series to which a given sample belongs (e.g., 40,841 stands for GSE40841). In addition, fields such as “Age” or “Sex” are included, describing the age of the sample's organism of origin and its sex (in 0–1 encoding; e.g., 0 stands for female and 1 stands for male). Moreover, the tissue of origin is described in two fields, “TissueID” which refers to the Brenda Tissue Ontology (BTO; Chang et al., 2014; e.g., 89 stands for BTO:0000089), and “TissueName” containing the name of the tissue from the BTO. The name of the origin species is included in the “Species” field (e.g., *Homo sapiens*). An example of the metadata format is shown in Supplementary Table 2.

### 2.3 Database design

BioVDB uses the current latest Qdrant version (1.7.2) to store vectorized data. Every sample in our database is represented by a vector of a length corresponding to the number of genes in a given GEO platform. Vector indexing is performed using the Hierarchical Navigable Small World Graph (HNSW), which is a default Qdrant graph-based indexing algorithm (Malkov and Yashunin, 2018). Metadata is stored along vectors as a payload, containing eight fields, covering information about GSM, GPL and GSE IDs, age, sex, tissue ID, tissue name, and species of the sample. Data is stored split between eight Qdrant collections, one per GEO platform. By default, the Euclidian distance is used as a metric for similarity search, however it is also possible to use dot product, cosine similarity or Manhattan distance.

### 2.4 Data structure analysis

To analyze the overall data structure and compare the raw and normalized data, we selected all samples from the two most common tissues in GPL570, blood, and breast. After filtering the samples, only those with ExperimentID and TissueName labels were included, resulting in a total of 27,348 samples (19,533 blood and 7,815 breast samples). Then only samples belonging to an ExperimentID that had two or more samples within were selected to allow batch correction. The final data set included only samples belonging to the top five most popular ExperimentIDs for blood and breast (5,573 samples in total). To process the data, we log transformed the raw gene expression values if not already log transformed, quantile normalized, and did batch effect correction based on ExperimentID, using pyComBat version 0.2.3 (Behdenna et al., 2023). To show the structure of the data and visually compare raw and processed data, we used Uniform Manifold Approximation and Projection (UMAP) version 0.5.3 (McInnes et al., 2020) and the Python library seaborn version 0.12.0 (Waskom, 2021).

### 2.5 Similarity search

A similarity search was performed for the 300 most similar samples to randomly chosen lung sample (GSM1001648) from GPL570. Euclidian distance was used as a distance metric, with hnsw_ef = 128 (value specifying the ef parameter of the HNSW algorithm). Processing and visualization were done using UMAP and seaborn, as mentioned above.

## 3 Results

### 3.1 Data overview

The BioVDB is a biological vector database that addresses current challenges in the field. Currently BioVDB contains microarray data that was downloaded from GEO. It stores 438,562 samples, covering eight platforms from GEO. There are in total 335,962 samples from *Homo sapiens* and 102,600 from *Mus musculus*.

### 3.2 Species distribution

**Figure 1:**
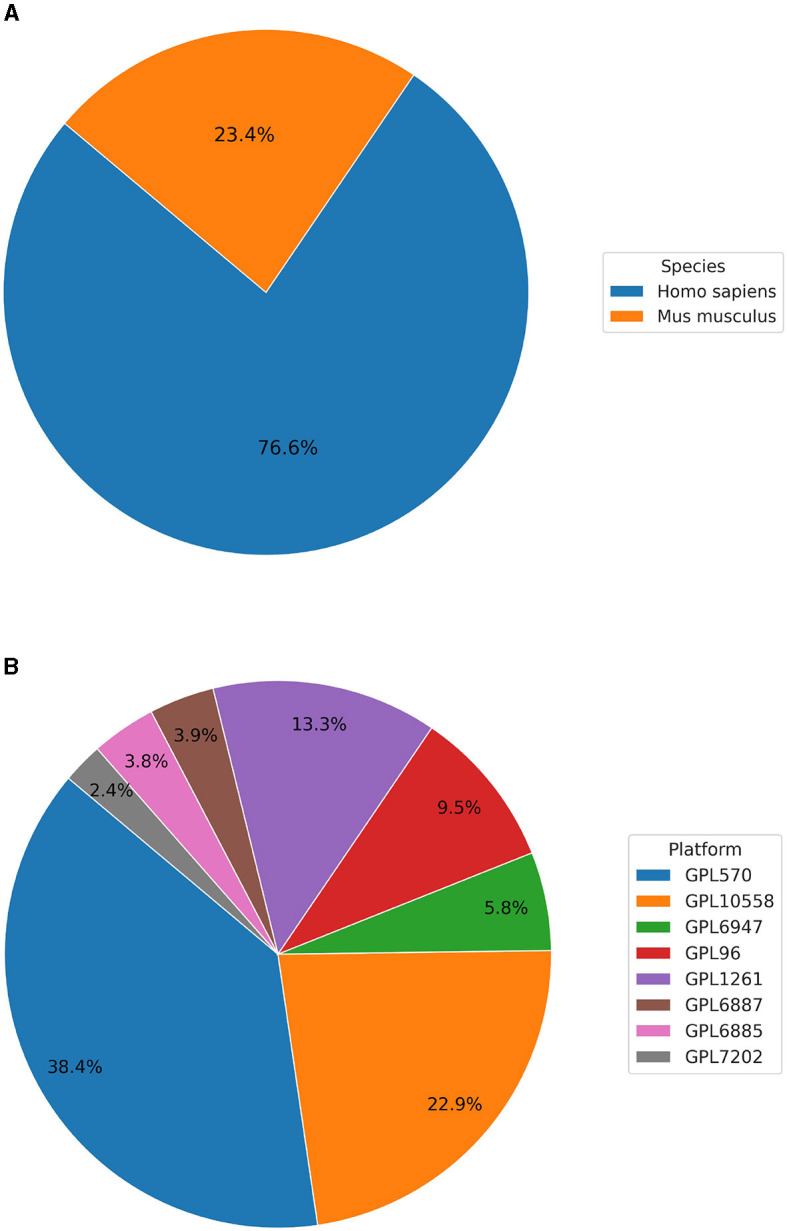
Distribution of samples by **(A)** species and **(B)** platform in the BioVDB.

Currently, BioVDB stores data from two species, *Homo sapiens* and *Mus musculus*, which make up the vast majority of samples in GEO (Figure 1). Samples from *Homo sapiens* account for 76.6% of all samples, with the rest coming from *Mus musculus* (Figure 1A). In the context of data distribution between platforms, BioVDB consists of 8 of the most abundant microarray platforms, with nearly 40% coming from GPL570 (Figure 1B). This platform, together with GPL10558, accounts for more than 61% of the total number of samples in BioVDB with the rest belonging to the other six platforms.

### 3.3 Tissue distribution

To allow for meta-analyzes of the samples by tissue type of origin, BioVDB also includes tissue name labels derived from BTO. The 10 most common tissues from GPL570 are shown in Supplementary Figure 2. Among the 196 unique tissues labeled for this platform, blood is the most abundant, with data derived from an ontology of tissues such as peripheral blood mononuclear cells (PBMCs) and dendritic cells (DCs), which account for the heterogeneous nature of this tissue. Blood accounts for twice as many samples as breast tissue, the next most common label. In addition, a comparison of the tissue distribution of the BioVDB samples and all samples for which labels were extracted is shown in Supplementary Figure 6.

### 3.4 Age distribution

Age distribution analysis was performed on all samples containing labels for age, sex, and tissue name (Supplementary Figures 3, 4). Of the total 21,853 samples, 10,165 were from females and 11,688 from males (Supplementary Figure 3). The average ages for female and male samples were 52.21 and 51.96, respectively. Among all tissues, samples were selected from the 10 most common ones, whose age distributions are shown in Supplementary Figure 4. Again, blood was the tissue containing the largest number of samples from the entire data set, and the samples taken from the human brain had the largest age range. In addition, the data set analyzed did not contain any breast-derived samples from men. A comparison of the age and sex distribution of the BioVDB samples and all samples for which labels were extracted is shown in Supplementary Figures 7, 8, respectively.

### 3.5 Structure of raw and normalized blood and breast samples

**Figure 2:**
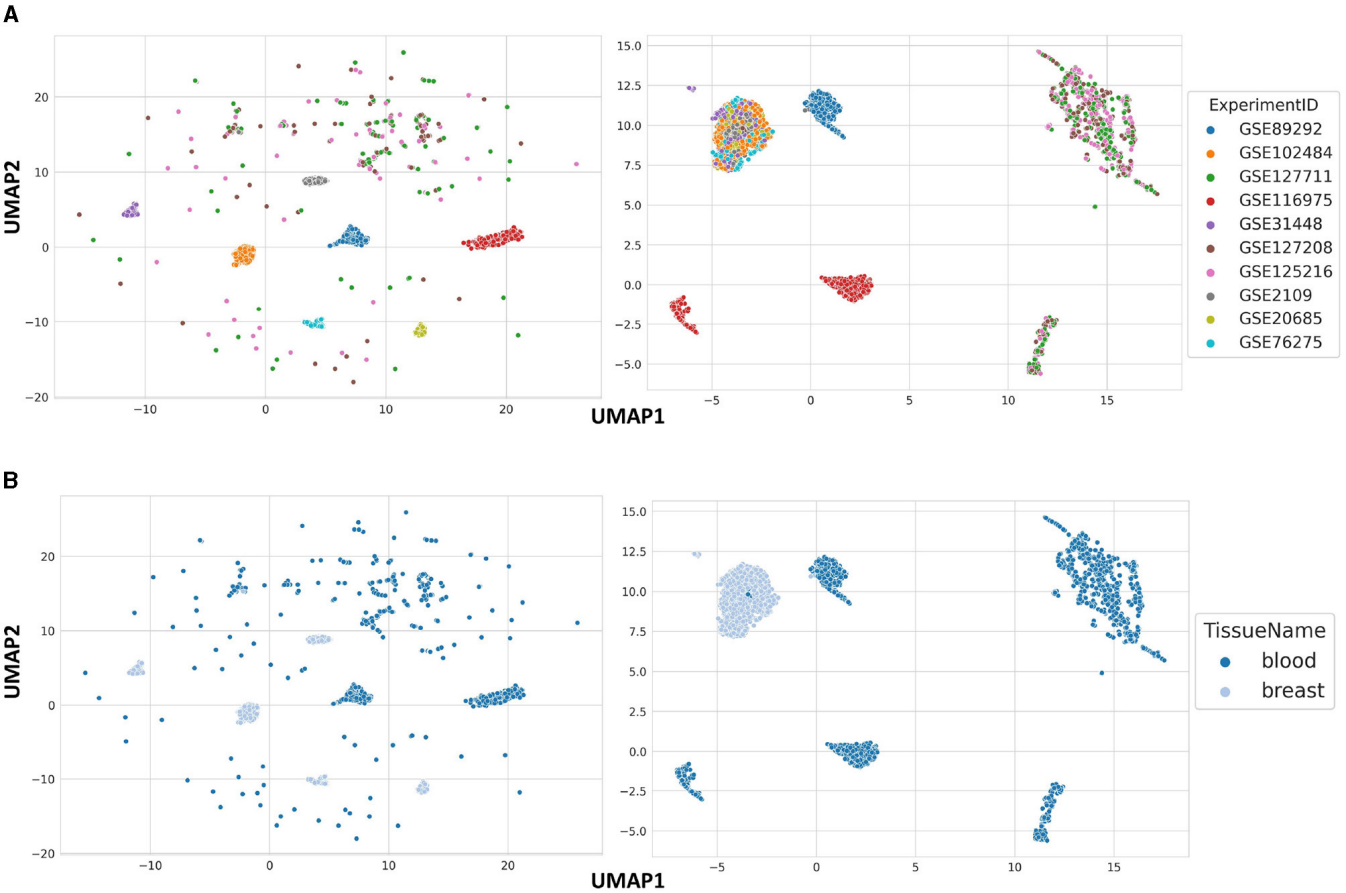
Data structure of raw **(left side)** and normalized **(right side)** blood and breast samples from GPL570, colored by **(A)** ExperimentID and **(B)** TissueName.

In order to show the general data structure, we used UMAP on raw and normalized blood and breast samples, two most common tissues in GPL570. The raw data are clustered based on ExperimentID, which was expected, given that it is the most influential batch effect in the dataset (Figure 2A). However, it is also worth noting that some of the samples in the analyzed dataset are sparsely scattered across the data space. The reason for this is the previously mentioned heterogeneous nature of blood, which is composed of different types of cells, which explains the kind of distribution shown in Figure 2B. After processing the data from samples from both tissues and correcting for batch effect, six clearly separated clusters stand out, based on the tissue type of origin of the samples, with the exception of a few samples (Figure 2B). This allows us to conclude that the stored data in BioVDB, after pre-processing, allows high-throughput analyzes of gene expression coming from different GEO platforms.

### 3.6 Label inference through similarity search

**Figure 3:**
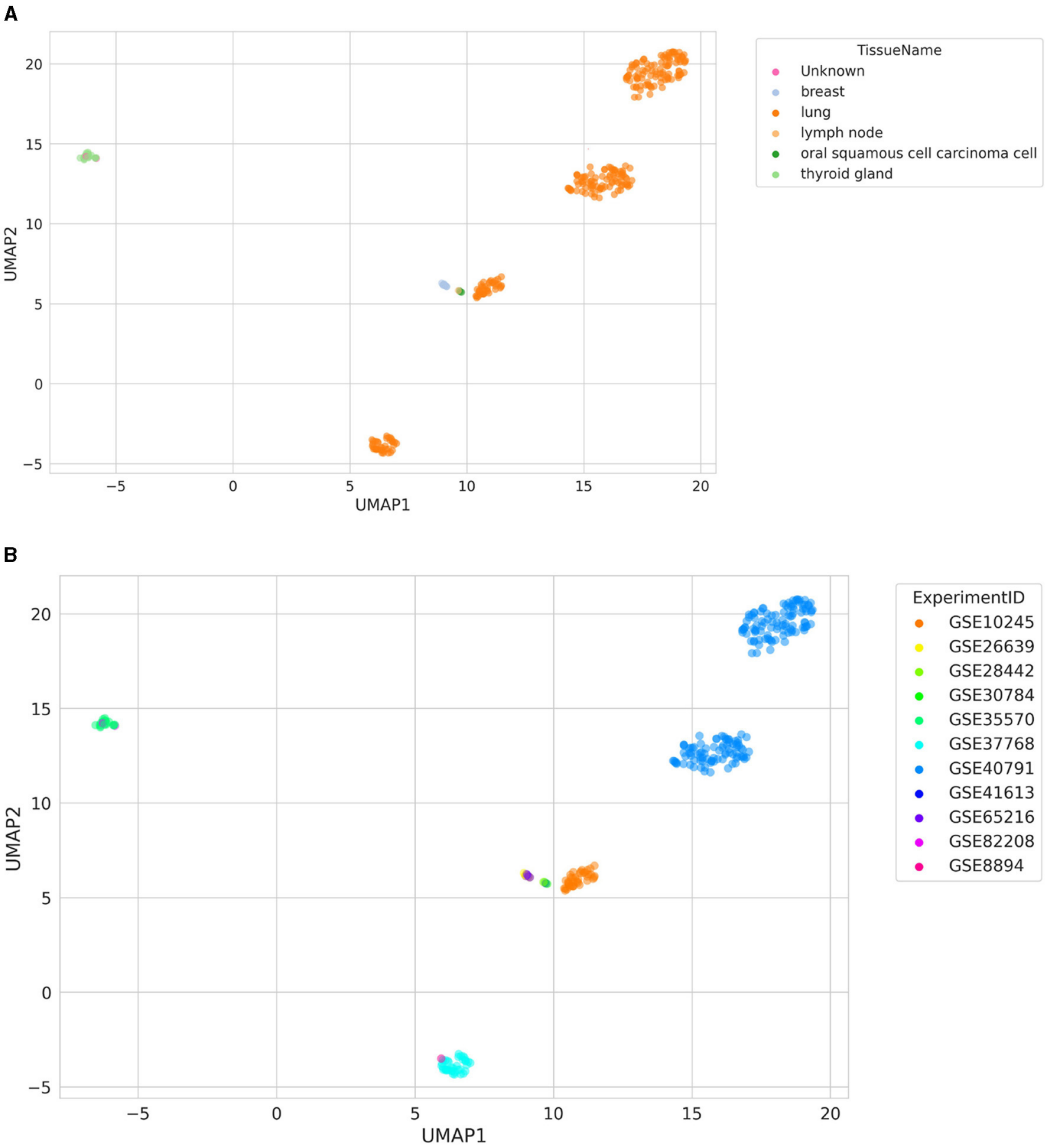
Distribution of samples retrieved using a similarity search colored by **(A)** TissueName and **(B)** ExperimentID. The “Unknown” label represents samples with missing TissueName label.

A visualization of the UMAP results of similarity search is shown in Figure 3. The searched samples spanned six different TissueNames, including “Unknown,” which symbolizes samples with missing tissue labels (GSM2186545 and GSM218653; Figure 3A). They were clustered together with samples from a thyroid gland (GSE35570), even though they came from a different ExperimentID (GSE82208; Figure 3B). In addition, it is worth noting that two separate clusters of lung samples are visible, originating from GSE40791. One of these clusters is lung adenocarcinoma samples, and the other is healthy lung samples, resulting in the clear separation of these clusters.

## 4 Discussion

### 4.1 Label accuracy and missing annotations

Currently, BioVDB stores age, sex, and tissue labels, extracted from GEO metadata, using ALE software. ALE uses a heuristic string matching algorithm that, with multiple matches for tissue, selects the most general node from BTO. This highly conservative approach provides relatively high annotation accuracy while causing generalization, which can be particularly noticeable in highly heterogeneous tissues like blood. This tissue is the most common in the GPL570 dataset we analyzed, as it includes samples such as PBMCs, DCs, or blood serum (Figure 3). At the same time, this affects the structure of the data, where some blood samples from the same experiment are sparsely scattered across space (Figure 2). Another aspect worth mentioning is the need to address the problem of distinguishing between healthy controls and case samples from the same experiment. This would make it possible to distinguish between cancer samples and healthy controls from the same tissue and experiment, such as in Figure 3, where one cluster was formed by lung adenocarcinoma samples and the other by healthy lung samples. This problem has been addressed by Lakiotaki et al. (2018), where the authors used predefined keywords for this purpose, although to distinguish samples in an experiment with multiple interventions it is insufficient and requires more sophisticated text mining solutions. Ultimately, this approach would allow differential gene expression (DGE) analysis between such cohorts. In addition to the quality and accuracy of the stored annotations, the sheer number of labeled samples in the BioVDB is also important. Of the 168,570 samples from GPL570 stored in BioVDB, only 21,853 have complete labels describing age, sex, and tissue (Supplementary Figure 3). This limits the amount of data available for analysis requiring all of these annotations and requires further work to fill in the missing labels.

### 4.2 Further applications

Despite the relatively large amount of metadata information stored in the BioVDB labels, it is possible to further expand them with disease-specific annotations and the aforementioned case vs control labels. Such information will allow robust comparative analyzes, making it possible to identify, for example, specific tissues where the difference in gene expression is largest in a given disease phenotype or to observe such changes in different age groups using the similarity search provided by Qdrant to do so. In the future, this could be useful in determining the brain regions where the greatest changes in gene expression occur in analyzes comparing healthy controls to samples from patients diagnosed with Alzheimer's or Parkinson's disease, which could yield new biological insights into the genetic mechanisms underlying neurodegenerative diseases. However, the most important aspect of such a research approach is to provide insight into global changes in gene expression, increased statistical power, and external reproducibility of analysis results compared to studies focusing only on, for example, the effect of one particular gene on a given phenotype.

The primary purpose of GEO was to store data from gene expression microarray experiments, but over the years, it also began to incorporate data from high-throughput sequencing experiments (e.g., RNA-seq), methylation arrays, and other types of biological data (Supplementary Figure 5). The next improvement of BioVDB will be to expand it to include those data that can be analyzed using, for example, multimodal machine learning algorithms. That will maximize the information in the analyzes contained in data of different modalities taking into account not only genetic but also epigenetic information, which may yield new conclusions of biological significance.

The current version of BioVDB contains data from eight microarray platforms, which are among the most abundant and popular containing data from *Homo sapiens* and *Mus musculus*. However, BioVDB will gradually be updated with data from other platforms. Eventually, BioVDB will be a database that is automatically updated weekly and will allow access to all samples from GEO. In addition, with future updates, more capabilities are planned to be added to BioVDB such as handling fusion genes and splice variants.

## 5 Conclusion

Here we present BioVDB, to the best of our knowledge, the first vector database of gene expression experiments. It stores 438,562 samples from eight microarray GEO platforms. With its standardized metadata format, the tool allows for meta-analysis of genomic data by selected cohorts, such as age, sex, or tissue of origin of the samples in question. Additionally, it provides a ready-to-use format for deep learning models due to encoded experimental gene expression values in vector form. What's more, it allows similarity search, which, as we presented, can help determine missing labels of the samples.

## Data availability statement

Publicly available datasets were analyzed in this study. This data can be found at: https://huggingface.co/collections/mwinn99/biovdb-658daf0c3ceccd00f3ad63a9. The BioVDB code can be found at: https://gitlab.com/wrenlab/biovdb.

## Author contributions

MW: Data curation, Formal analysis, Investigation, Software, Visualization, Writing - original draft, Writing - review & editing. CB: Software, Writing - review & editing. HP: Formal analysis, Validation, Visualization, Writing - review & editing. CG: Data curation, Software, Writing - review & editing. JW: Conceptualization, Funding acquisition, Methodology, Project administration, Resources, Supervision, Writing - review & editing.
