# Supplementary material for: BioVDB: biological vector database for high-throughput gene expression meta-analysis
Source: Front Artif Intell. 2024 Mar 8;7:1366273. doi: 10.3389/frai.2024.1366273 (PMC10957786; doi:10.3389/frai.2024.1366273)
Supplement: Supplementary file 1 [file Data_Sheet_1.PDF]

Supplementary Material

1 SUPPLEMENTARY TABLES AND FIGURES

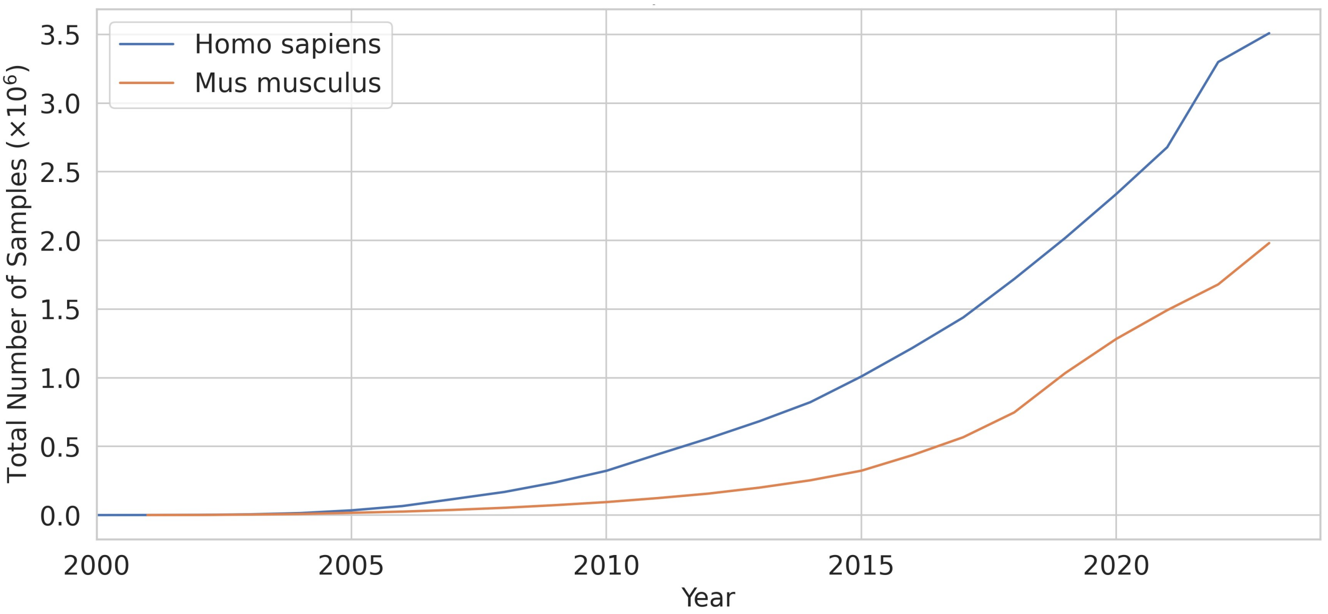

Figure S1: Increase in the total number of samples in GEOmetadb over time. The number of samples in units of 10^6 refers to the number of GSM samples from GEO. Data downloaded and analyzed using software from R package GEOmetadb Zhu et al. (2008).

Table S1. Number of samples per type in GEOmetadb.

Type	Count
SRA	4,179,485
RNA	1,853,940
Genomic	572,439
Protein	38,290
Other	10,890
Mixed	4,978
SAGE	1,771
MPSS	207
SARST	9
**TOTAL**	6,662,009

Table S2. Example of sample's metadata in BioVDB. PlatformID represents GEO GPL platform, ExperimentID stands for GEO GSE series. TissueID and TissueName fields contain information about tissue of origin ID from BTO Chang et al. (2014) and name its name, respectively.

GSM	PlatformID	ExperimentID	Age	Sex	TissueID	TissueName	Species
GSM1003121	10558	40841	58.0	1.0	89.0	Blood	Homo sapiens

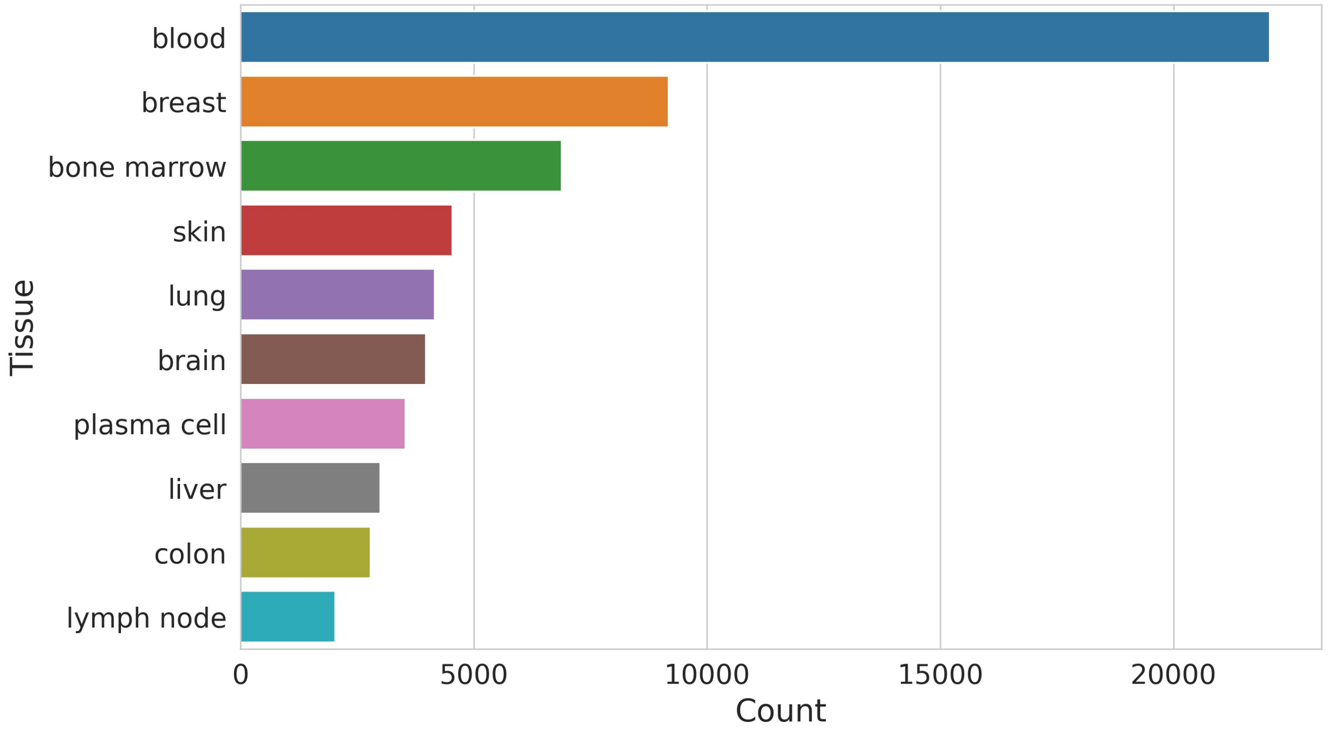

Figure S2: Number of samples for each of the top 10 most common tissues in GPL570.

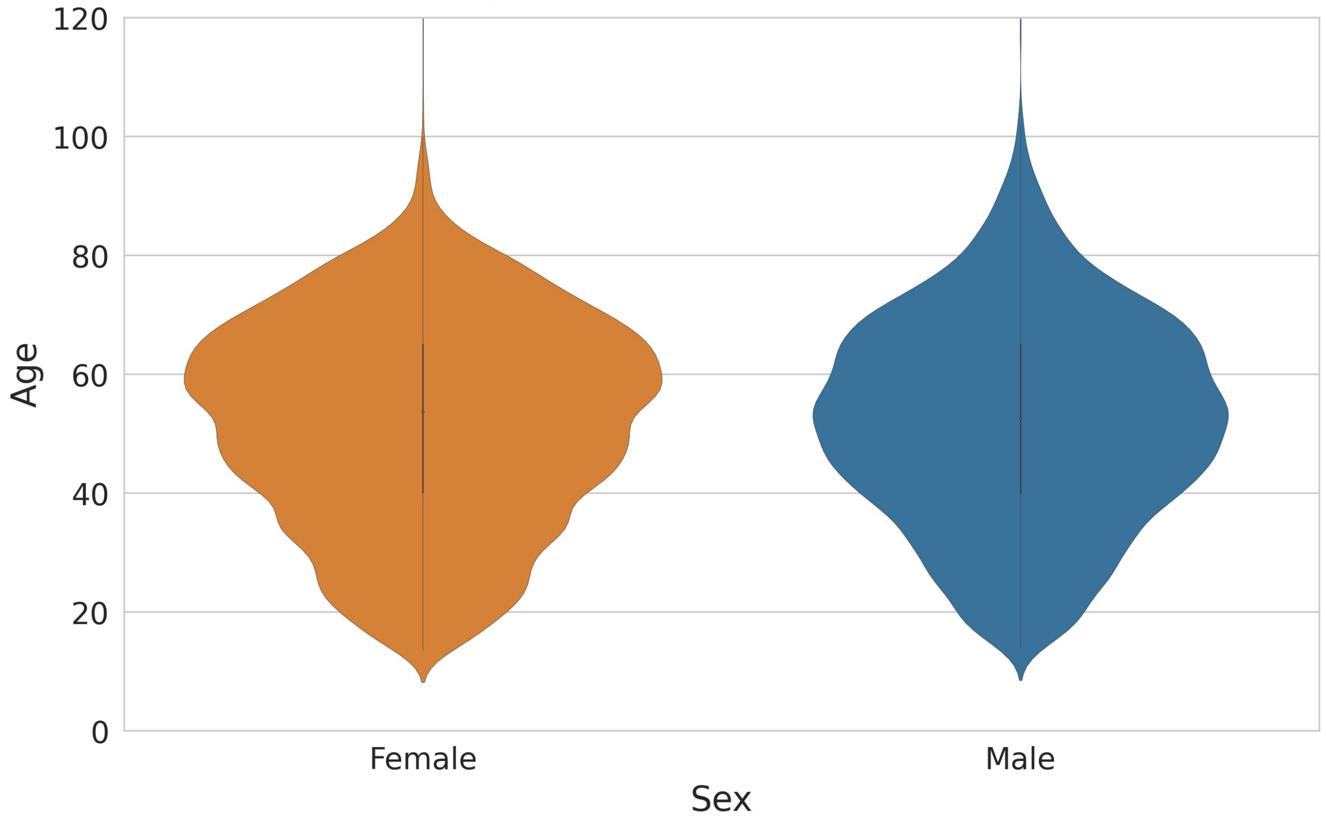

Figure S3: Age distribution of GPL570 samples by sex.

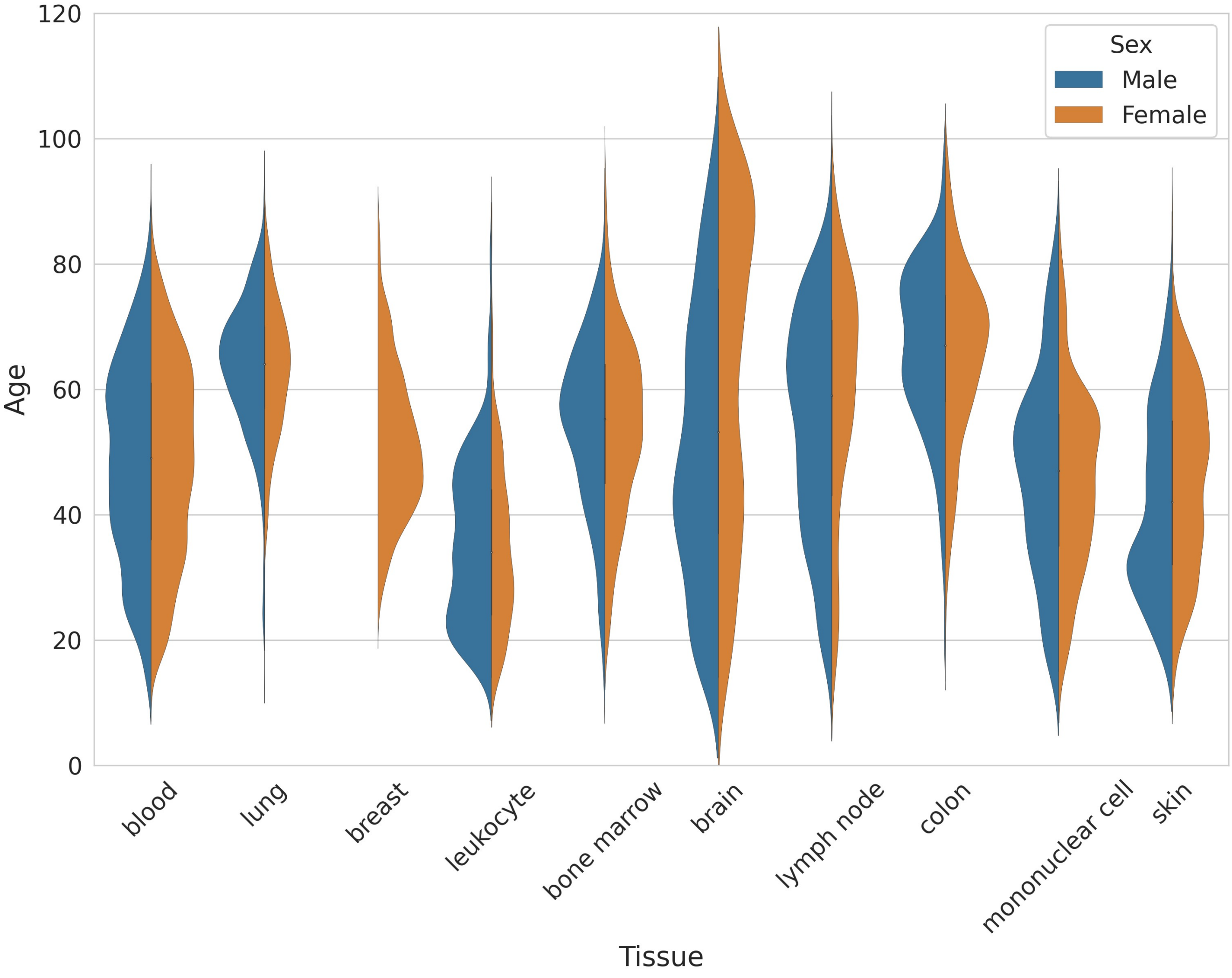

Figure S4: Age distribution of GPL570 samples by tissue.

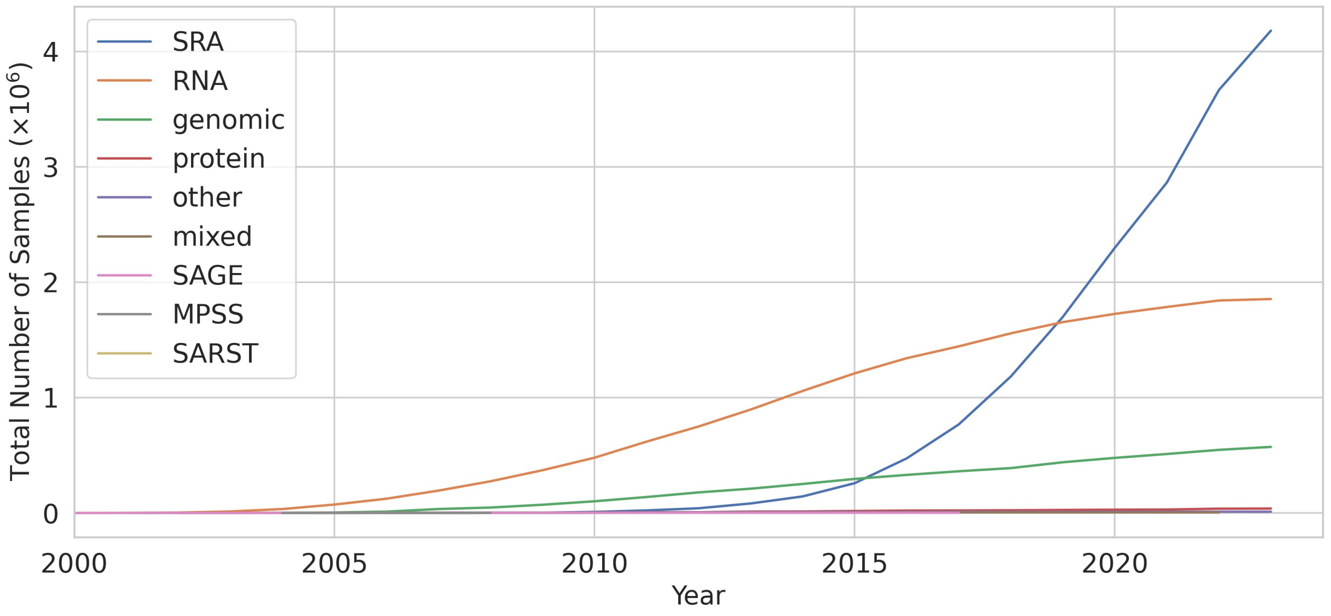

Figure S5: Change of the number of samples in GEOmetadb by type. The number of samples in units of 10^6 refers to the number of GSM samples from GEO. Data downloaded and analyzed using software from R package GEOmetadb Zhu et al. (2008).

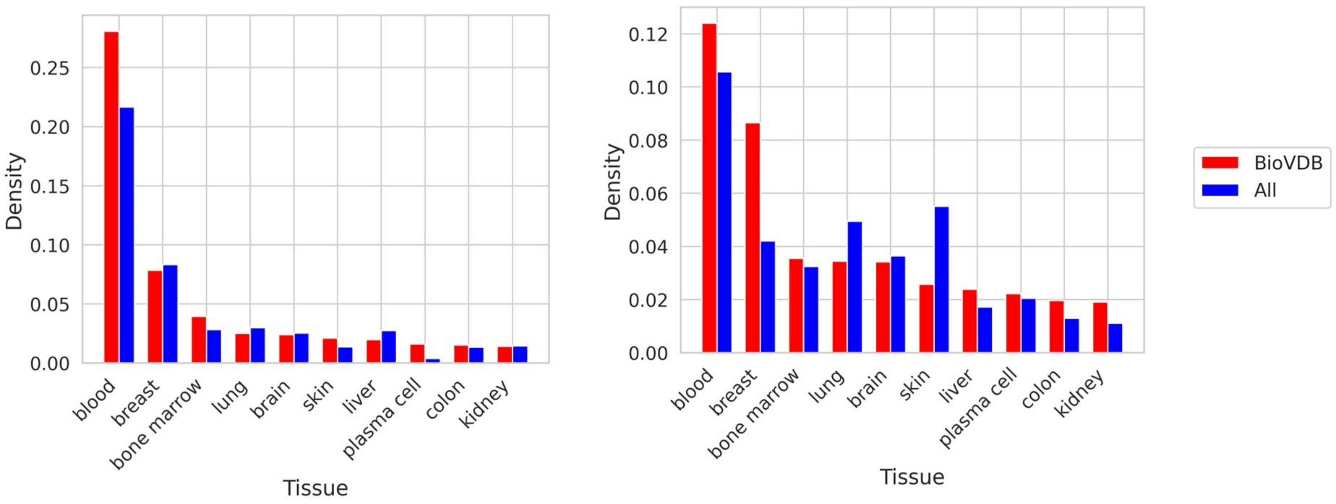

Figure S6: Distribution of *Homo sapiens* (left) and *Mus musculus* (right) samples by tissue. Only the top 10 most common tissues are shown. The distribution of BioVDB samples is shown in red, while the distribution of all samples (*Homo sapiens* n=1,881,470; *Mus musculus* n=381,498) whose labels were extracted is shown in blue.

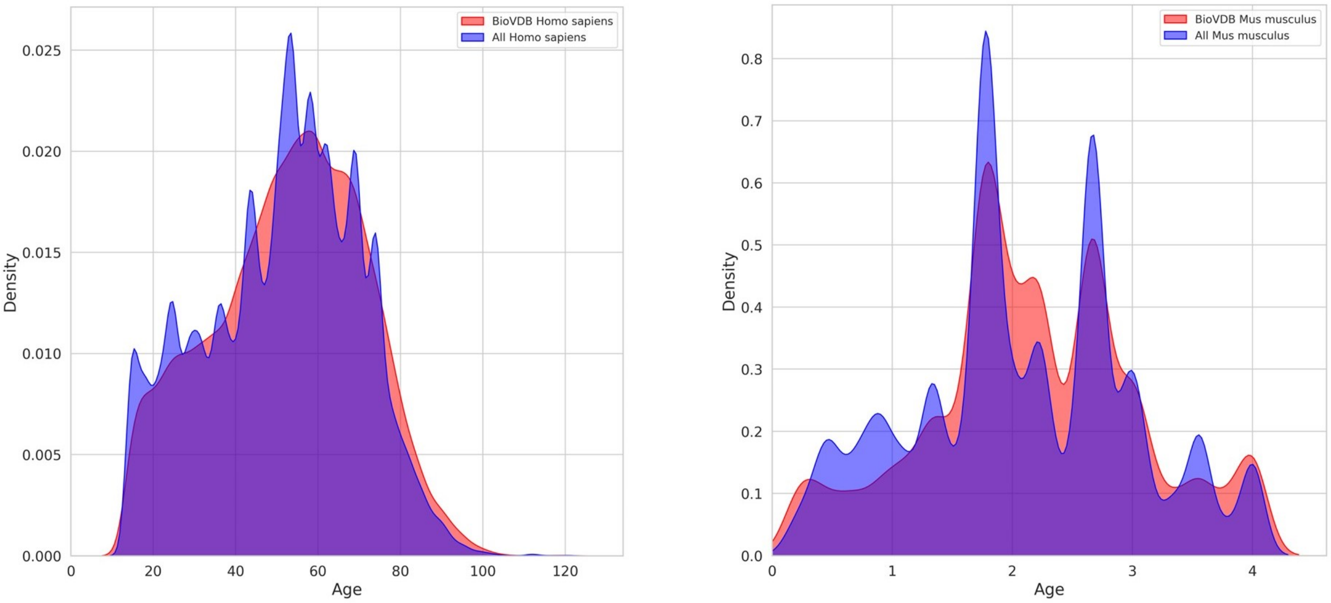

Figure S7: Distribution of *Homo sapiens* (left) and *Mus musculus* (right) samples by age. The distribution of BioVDB samples is shown in red, while the distribution of all samples (*Homo sapiens* n=1,881,470; *Mus musculus* n=381,498) whose labels were extracted is shown in blue.

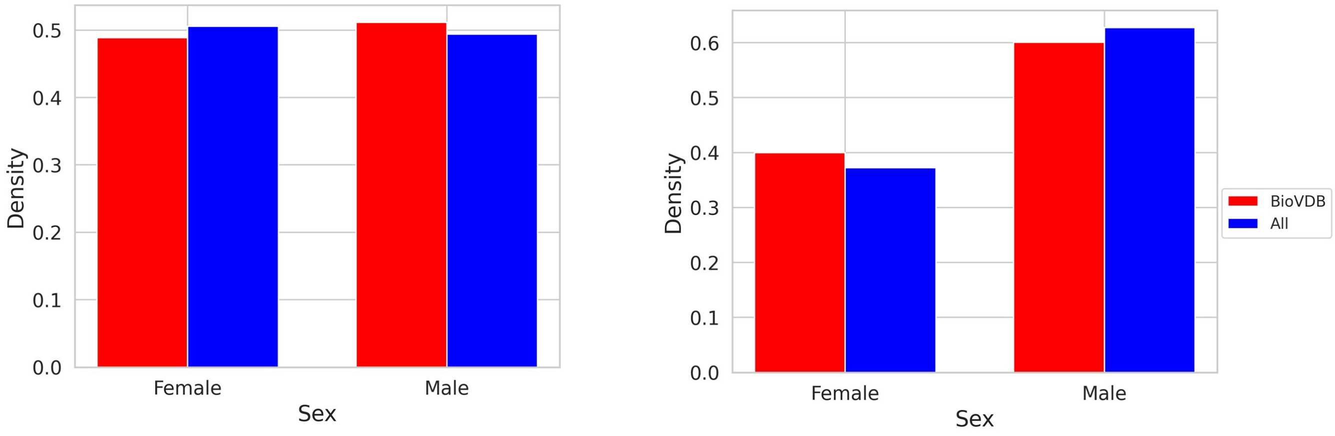

Figure S8: Distribution of *Homo sapiens* (left) and *Mus musculus* (right) samples by sex. The distribution of BioVDB samples is shown in red, while the distribution of all samples (*Homo sapiens* n=1,881,470; *Mus musculus* n=381,498) whose labels were extracted is shown in blue.

REFERENCES

Chang, A., Schomburg, I., Placzek, S., Jeske, L., Ulbrich, M., Xiao, M., et al. (2014). Brenda in 2015: exciting developments in its 25th year of existence. *Nucleic acids research* 43, D439–D446. doi:https://doi.org/10.1093/nar/gku1068

Zhu, Y., Davis, S., Stephens, R., Meltzer, P. S., and Chen, Y. (2008). Geometadb: powerful alternative search engine for the gene expression omnibus. *Bioinformatics* 24, 2798–2800. doi:https://doi.org/10.1093/bioinformatics/btn520
